# Drivers and barriers to achieve a circular economy of small-IT: Case study from the city of Porto

**DOI:** 10.1177/0734242X21994917

**Published:** 2023-09-08

**Authors:** Marisol Espino Penilla, Asmita Dubey, Maurice Prot, Evi-Mara van Beekhuizen, Elie Deeb, Soraia Taipa, Telmo Machado

**Affiliations:** 1 Wageningen University & Research, Beesd, Netherlands; 2 Research & Innovation Department, LIPOR – Intermunicipal Waste Management of Greater Porto, Portugal

**Keywords:** E-waste, drivers and barriers, small-IT, circular economy, collection, recycling behaviour

## Abstract

Advances in digital electronics delivered small and portable gadgets, changing human interface with technology. Demand for new small devices of Information and Telecommunication Technology (small-IT) that have a short lifespan, like smartphones and laptops, creates flows and accumulation of electronic resources. These include precious metals that show potential for Urban Mining and Circular Economy. To find out the extent of setting up an improved recycling, reuse and repair system, data collection was conducted through surveys, bin observations and social experiments. These methods enabled us to analyse stocks and flows, identify behavioural practices and map collection infrastructure. About 80% of domestic small-IT stocks are hibernated, meaning they could be directly reused or easily repaired. Results show four barriers that keep citizens from handing in their unused small-IT: Devices are kept as back-up, they contain sentimental value, citizens are suspicious of post-collection data confidentiality issues and there is a perceived high effort to recycle. Drivers to enhance circularity are: improving e-waste infrastructure, introducing economic incentives and raising awareness on environmental impacts of hibernated stocks. A more trustworthy and legitimate management system is expected to deliver safety and confidentiality of personal data and provide the quality that fits the expectations of citizens of a proper place to dispose of their valuable items.

## Introduction

Small devices of Information and Telecommunications Technology (small-IT), like smartphones and laptops, are usually replaced by users with newer, more powerful models about every 18 months (United States Geological Survey, 2011; ProSUM Project, 2017; Allied Market Research, 2015). Due to the rapid microchip development, the lifespan of electronics has reduced increasingly (Moore, 1965; United States Geological Survey, 2011). This has led to a growing flow of e-waste (Baldé et al., 2015) and a build-up of a hibernated stock of small-IT in homes and shops (ProSUM Project, 2017), which include devices that are working or easily fixable but are no longer in use in the premises of their owner.

Considering data from all EU 28 Member States plus Switzerland and Norway, the stock (in use or hibernated) goes up to nine products per person with an average weight of 12 kg/person, showcasing the potential for Urban Mining (ProSUM Project, 2017). Global electronic waste was expected to increase to a staggering 52.2 million metric tonnes, or 6.8 kg per inhabitant, in 2021 (Baldé et al., 2017). Electronic waste is now often referred to as the fastest growing solid waste stream and its management has proven to be unbelievably challenging (Parajuly et al., 2019). Globally, only 20% of the electronic waste that makes its way out of a home gets recycled, and the rest is thrown into the residual waste and is likely to be incinerated or landfilled (Baldé et al., 2017). Decision-makers and waste managers need further knowledge on how to engage citizens with recycling, reuse and repair of small-IT, especially in urban areas (Allied Market Research, 2015; Cucchiella et al., 2015).

The current lifecycle of electronics poses serious threats to the environment. The production process of many small electronic devices requires metals and other materials acquired from environmentally damaging extractive activities (Bekaroo et al., 2018; McKinsey, 2020). On average, the emissions of the production phase of electronics account for 70% to 80% of the footprint of personal electronic devices (Oeko-Institut e.V., 2016). Adding to the environmental impacts of high demand and production, when electronics’ use comes to an end, recycling rates are low and hibernated stock is high. Reusing resources contained in e-waste and hibernated stock of small-IT could avoid the extraction of raw materials, thus reducing the environmental impacts of electronics. Circularity in electronic devices is therefore important, as it helps mitigate negative environmental impacts and can bring added economic and social benefits (Allied Market Research, 2015; Greenpeace, 2017). Disclosing what could motivate citizens to engage in recycling, reuse and repair of small-IT is imperious to increased sustainability in urban areas.

Porto, the second largest city of Portugal, has been increasing the e-waste recycling rates with the implementation of programmes by the local waste management organisation – LIPOR. Nevertheless, when compared to the number of new electronics sold, the number of materials sent for recycling is still far from reaching its recovery potential. Depending on the conditions and obsolescence of hibernated small-IT, some of them can be directly reused without the need of fixing them, others can be repaired and reused after or otherwise recover its materials for recycling. Using social investigation methods and material flow analysis, research was conducted to assess the potential for Urban Mining in Porto and to identify citizens’ behaviours and motivations that need to be properly addressed in a new strategy to enhance the circularity of small-IT.

## Materials and methods

The study focused on the stock and flow analysis of small electronics such as smartphones, laptops, tablets, e-readers, digital cameras, gaming consoles and media players. Data collection for the analysis was achieved using three data collection tools: (a) surveys (b) audits on e-waste collection points and (c) experiments on alternatives to the current e-waste system. Data were collected in the areas of: União de Aldoar, Foz do Douro e Nevogilde (high income, business activities); União de Lordelo do Ouro e Massarelos (mixed income, universities); Paranhos (middle income, students and universities); União de Cedofeita, Miragaia, S.Nicolau, Sé, St Ildefonso, Vitória (mixed income, commercial activities) and Bonfim (low income, commercial activities).

### Surveys

**Figure 1.**
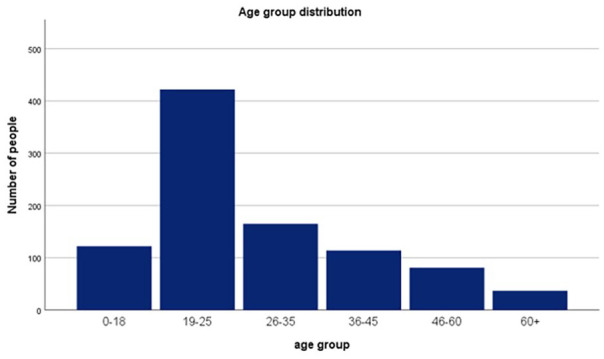
Citizen survey participants age group distribution.

Two surveys were developed to collect quantitative and qualitative data on small-IT from different perspectives: one for citizens and another for shop owners. The survey designed for citizens searched for data related to the acquisition and replacement of small-IT on a 3-year period, overall condition of the equipment after use, awareness and social practices and preferences on delivering unused small-IT (Table 1). Convenience sampling was used and respondents were required to live in Porto and be over the age of 13. Subsequently, a total of 963 citizen surveys were collected (Figure 1). The survey designed for electronic, second-hand and repair stores had the aim to gain insight into the return policy of small-IT and investigate possible synergies that could be created in collaboration with LIPOR. A total of 33 stores collaborated with the study. IBM SPSS Statistics (Version 25) was used to analyse the data.

**Table 1.** Citizen survey design to identify social practices, demographics, and to measure stock and flow for small-IT in Porto.

Category	Question objective	Question description
Stock and flows analysis	Current stock of hibernated small-IT and the overall condition of the devices after use	Table relating the amounts of each type of electronic device they currently had but were not in use, and its condition
	Inflow: Acquisition of small-IT on a 3 year period	Table relating the number of devices they had acquired over the last 3 years and the condition of acquisition (new or second hand)
	Outflow: Replacement of small-IT on a 3 year period	Table relating the amount of devices replaced in the last 3 years and information about what they had done with it
Social practices	Motives for current outflow practices	Multiple choice for motives, depending on the responses in the outflow table
	Environmental awareness and willingness to engage with recycling	Four-point Likert scale, from ‘strongly disagree’ to ‘strongly agree’
	Preferences on delivering unused small-IT, incentives and stakeholder responsibilities	Checkboxes with several answers possible
Characteristics respondents	Perception of current small IT recycling efforts	Awareness of IT recycling campaigns and satisfaction with current efforts. Multiple choice questions
	Demographic information	Parish of Residence, age, occupation and household composition

### Audits on e-waste collection points

The European Recycling Platform (ERP) website shows 18 locations of small-IT collection points in the research area (Rede Depositrão • ERP Portugal. n.d). Visits to announced locations were performed to audit the current infrastructure, verify the accuracy of the information provided to citizens, its maintenance, visual aspects, sizes and accessibility. During these audits several observations were conducted on the status of the disposed equipment.

### Experiments on alternatives to the current e-waste system

To investigate collection method’s viability and efficiency, two experiments were designed. One to test if door-to-door collection would be a viable strategy and the second to measure the effectiveness of a drop-off collection point at an area in which people will return to. The door-to-door collection experiment started by distributing flyers to 200 households in the research area, giving a 4 days’ notice for researchers to return and collect hibernated small-IT. For the second collection experiment, a collection point was set up inside a festival area (NOS Primavera Sound between 6th and 8th June 2019), and the study was explained to people approaching the stand. Bags were distributed for people to bring their old gadgets back to the collection point the following day.

## Results and discussion

The collected data were used to analyse the stocks and flows of small-IT and observe citizens’ behaviour towards these devices. As convenience sampling was used, our sample is unrepresentative of Porto’s population. Even though the survey was distributed in different socioeconomic areas of the city, 75% of respondents were under the age of 36 (Figure 1), while the average age in Porto is 47.7 (UrbiStat S.r.l, 2020). Further research in older age groups is therefore recommended.

### Stock and flows analysis

**Figure 2.**
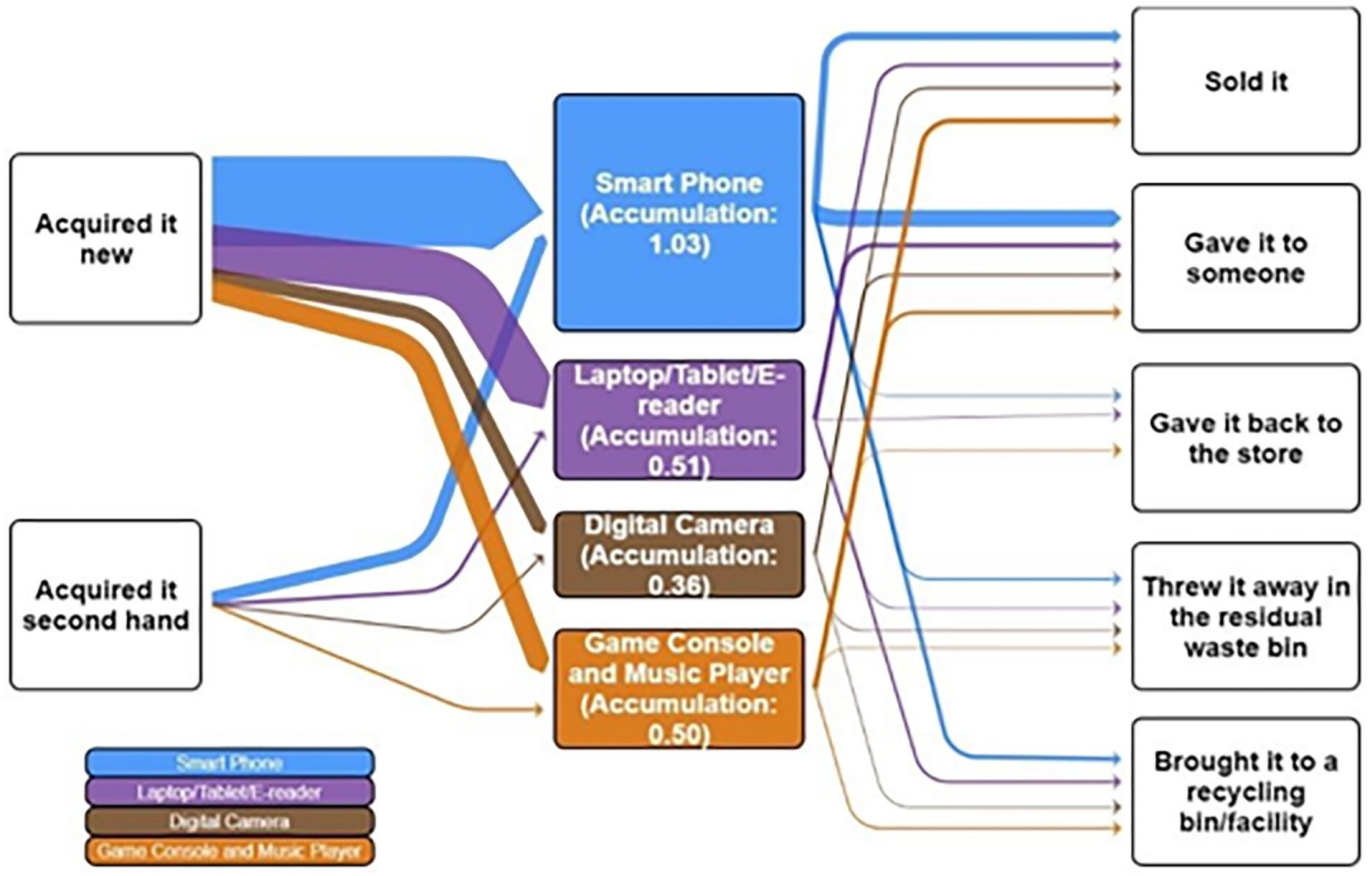
Illustration of the pathway of average small-IT per capita in Porto per 3 years.

Inflow’s results show first-hand products as the primary choice when acquiring new small-IT devices (Figure 2). Only 12% comes from second-hand shops, exemplifying the small contribution of the reuse market. The outflow, which measured how small-IT leaves the households, shows that recycling rates are low and that some devices still end up in the mixed waste. Furthermore, 75% of respondents keep their old phones at home, showcasing that accumulation is the most frequent behaviour. A detailed analysis of accumulated data, presented that 59% of the small-IT left in houses are directly reusable and 21% are repairable, presenting a potential for improving circularity of small-IT, regarding reuse, repair and recycling.

The stocks and flows analysis confirmed that accumulation represented citizens’ most regular choice for unused small-IT. Discovering and understanding drivers and barriers related to unused small-IT behaviour is crucial to remove potential hindrances or increase stimulating factors for an effective circular economy, in particular the one related to accumulation. For that reason, the behavioural patterns towards the accumulation of small-IT (barriers to small-IT circularity) and drivers to increase small-IT circularity are detailed in the following paragraphs.

### Barriers to small-IT circularity (reasons to accumulate hibernated small-IT)

#### Use as backup

**Figure 3.**
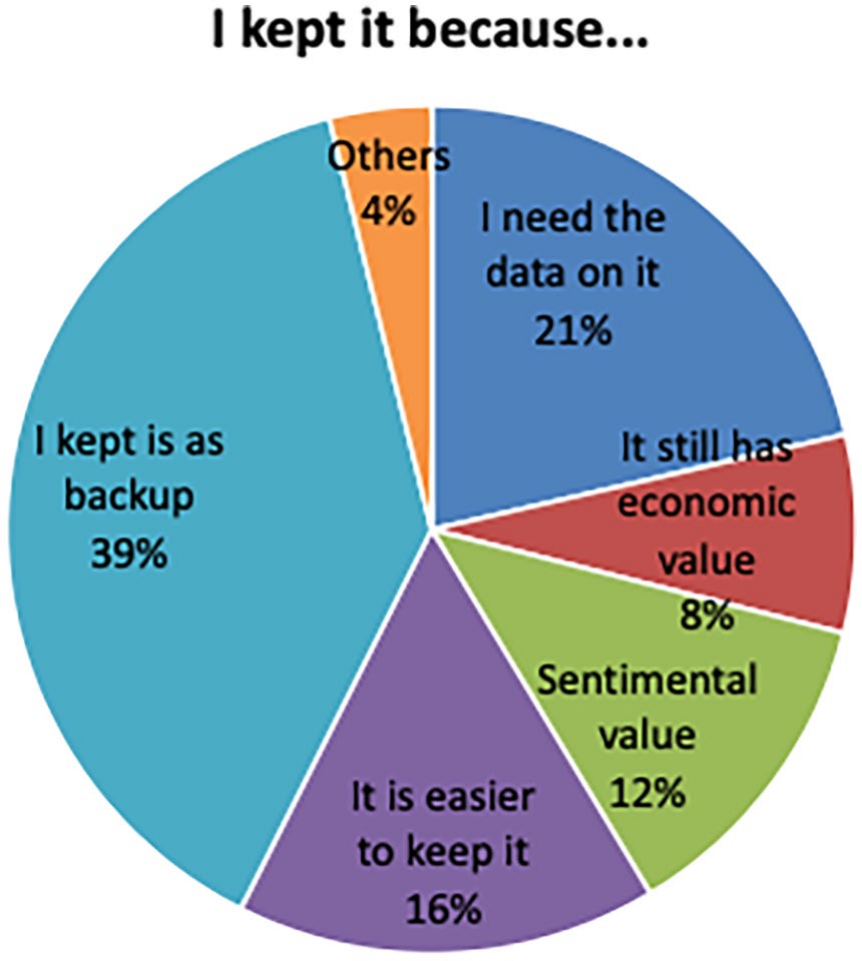
Reasons to accumulate hibernated phones.

Surveys indicated that the main reason why people keep their small-IT at home is that they would like to keep it as a backup (39% of respondents) (Figure 3). However, most citizens admitted that they never used the devices again, not even for this purpose.

#### Sentimental value

Sentimental value was recognized by 12% of respondents as the main reason to keep their small-IT (Figure 3). Portable electronic devices, particularly smartphones and digital cameras, are used on a daily basis and are frequently used to preserve long-lasting memories of important moments.

#### Data management and confidentiality

Small-IT are frequently used to store important and sensitive information. About 21% of the survey respondents referred to the stored relevant information in their old devices as the main reason for keeping them (Figure 3). During the citizen interviews, a concern about what happens to personal data was also mentioned. Confidentiality and reliable data erasing systems are missing in the current small-IT waste management system.

#### High effort to recycle

Recycling is perceived as requiring a high effort, which makes people less likely to hand in their small-IT (Figure 3). About 16% of the survey respondents indicated the easiness of keeping their devices at home was the main reason to do it. Survey analysis showed a 10% decrease in recycling rates for respondents who had indicated that recycling was too much effort. The survey results point out that 60% of Porto citizens do not know where to find recycling bins, more than half stated their neighbourhoods lacked engagement in the recycling of electronics, and 25% of Porto citizens were unaware of recycling as an offered option for small-IT disposal.

From the 18 bins indicated by ERP in the research area only three were found where indicated and six were found randomly in stores. The current infrastructure has dispersed bins in parking lots and supermarkets which are easily missed.

### Drivers to increase circularity of small-IT

Citizens’ preferred delivery options were analysed by the surveys, observations and experiments.

#### Improving e-waste infrastructure

Improving the infrastructure to collect e-waste can reduce the citizens’ perceived effort to recycle. With the experiments and surveys, citizens’ preferred options and behaviour were analysed to disclose if the first option would be a door-to-door or a drop-off system.

**Figure 4.**
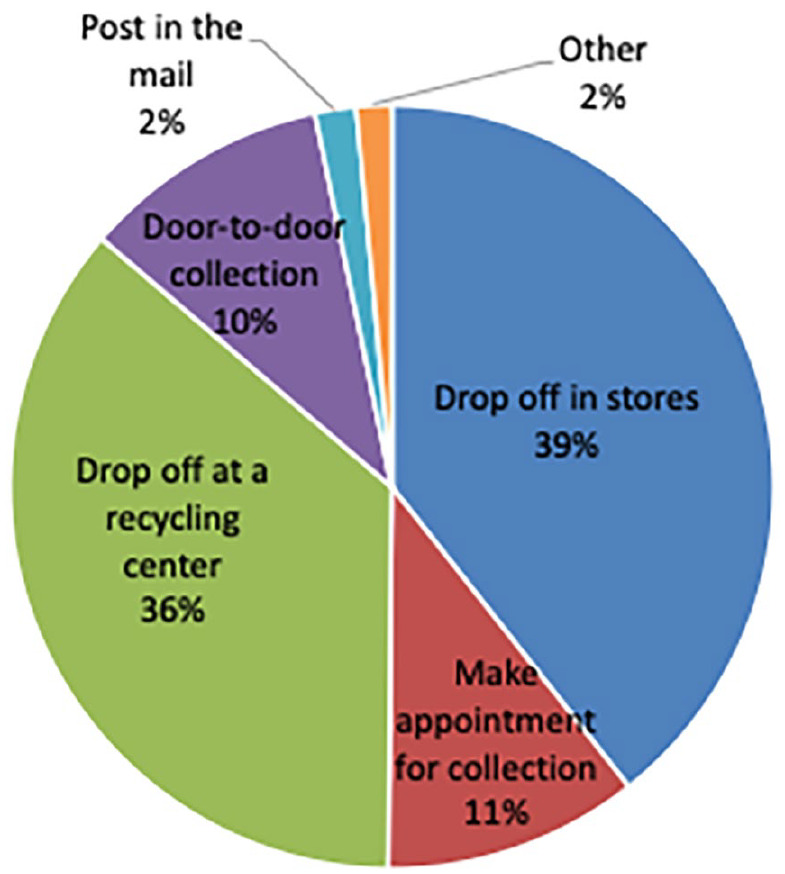
Preferred method of e-waste collection.

Only 2 out of the 73 households that opened the door on the collection date handed in their small-IT. The collection bin from the festival received small-IT from approximately 7% of the participants. The low participation rates in the collection methods from the experiments were in line with the results of the surveys, showcasing that traditional places, like stores are considered the most convenient. About 39% of the respondents selected drop-off at electronic stores, and 36% of the respondents selected drop-off at a recycling centre as a preferred method of collection (Figure 4).

#### Incentives

**Figure 5.**
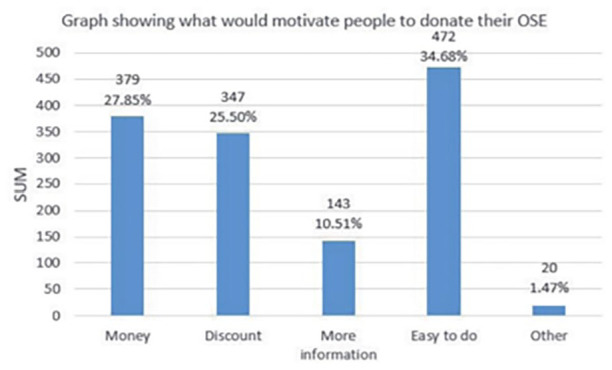
Incentives for Obsolete Small Electronics (OSE), which has been replaced with hibernated small-IT donation.

Data about incentives and citizens’ motivations to donate their unused small-IT showed that most of the respondents felt that money or vouchers were most likely to entice them to hand in their old electronic devices. It was also observed that respondents under the age of 25, mostly students, preferred money and vouchers as incentives (Figure 5).

#### Raise awareness on environmental impacts of hibernated stock of resources

Results indicated that there is no significant correlation between being environmentally aware, the willingness to recycle, and the actual act of recycling. Seventy percent of the respondents were found to be environmentally aware, yet only 10% recycled. However, conversations between researchers and respondents at the festival indicates that addressing the knowledge gap about small-IT impacts made people reconsider their attitude towards recycling, reuse and repair.

A study by Welfens et al. (2016) identified sentimental value, perceived effort of recycling, knowledge generation, improved infrastructures and economic incentives as relevant factors influencing recycling of small-IT. Darby et al. (2005) found that consumers and decision-makers do not prioritise the reuse and recycling of small-IT. The results of our research can be taken as a case study that reinforce the findings of previous research.

## Conclusion

A combined analysis of the gathered information showed that most people keep their small-IT even when they are not in use. With 75% of respondent’s small-ITs being kept and 80% of that hibernated stock being either still working or easily fixable, results show there is potential for urban mining in Porto. To be able to unlock this potential, it is essential to recognize the factors that influence people’s decisions of what to do with their small-IT after they are done using them. Citizens behaviour was thus analysed to identify both drivers and barriers that can increase reuse and recycle rates of small-IT, contributing to circularity.

The results showed that concerns for data confidentiality, sentimental attachment to the objects, the possible reuse as backup and perceived high effort for recycling are the most relevant barriers for the circularity of small-IT in Porto. Drivers for improving small-IT circularity were identified not only by citizens as incentives (in the form of money and vouchers) but also as donations for social causes. Increasing awareness on environmental impacts of hibernated stock of resources, and increasing trust through transparency were also mentioned as a way to reach relevant values of small-IT recycling or reuse.

Furthermore, e-waste collection infrastructures could benefit from including users’ concerns and preferences in their design and locations. In Porto, increasing drop-off options at electronic stores or recycling centres was the preferred option. The strong preference for a drop-off system instead of a collection one, indicates users rather have the liberty of delivering their small-IT whenever it is more convenient for them. Further research is needed to determine other reasons why this option was chosen.

These barriers and drivers present a challenge to waste managers, and recyclers as these entities will have to address not only technical issues of the system but also behaviour issues. The identified barriers and drivers are highly social, including a human aspect which is frequently overlooked in the design of recycling campaigns. Recognizing the social context and the intangible values of small-IT and including them in the overall design of the strategy to engage in urban mining, from the awareness campaigns, to the collection strategies, and the post-collection plans, is key for its success.
